# Sensor Network-Based and User-Friendly User Location Discovery for Future Smart Homes

**DOI:** 10.3390/s16070969

**Published:** 2016-06-27

**Authors:** Ehsan Ahvar, Gyu Myoung Lee, Son N. Han, Noel Crespi, Imran Khan

**Affiliations:** 1 Institut Mines-Telecom, Telecom SudParis, Evry 91011, France; son.n.han@outlook.com (S.N.H.); noel.crespi@telecom-sudparis.eu (N.C.); 2 Liverpool John Moores University, Liverpool L3 3AF, UK; G.M.Lee@ljmu.ac.uk; 3 Schneider Electric Ind., Corporate Research Center, Technology, Grenoble 38000, France; imran@ieee.org

**Keywords:** user location discovery, sensor network, user friendly, fuzzy set, smart homes

## Abstract

User location is crucial context information for future smart homes where many location based services will be proposed. This location necessarily means that User Location Discovery (ULD) will play an important role in future smart homes. Concerns about privacy and the need to carry a mobile or a tag device within a smart home currently make conventional ULD systems uncomfortable for users. Future smart homes will need a ULD system to consider these challenges. This paper addresses the design of such a ULD system for context-aware services in future smart homes stressing the following challenges: (i) users’ privacy; (ii) device-/tag-free; and (iii) fault tolerance and accuracy. On the other hand, emerging new technologies, such as the Internet of Things, embedded systems, intelligent devices and machine-to-machine communication, are penetrating into our daily life with more and more sensors available for use in our homes. Considering this opportunity, we propose a ULD system that is capitalizing on the prevalence of sensors for the home while satisfying the aforementioned challenges. The proposed sensor network-based and user-friendly ULD system relies on different types of inexpensive sensors, as well as a context broker with a fuzzy-based decision-maker. The context broker receives context information from different types of sensors and evaluates that data using the fuzzy set theory. We demonstrate the performance of the proposed system by illustrating a use case, utilizing both an analytical model and simulation.

## 1. Introduction

The term smart home describes homes equipped with intelligent technologies (i.e., context-aware systems and services) that generate an added value for the user. Smart homes are usually privately-used homes (e.g., residential houses and apartments) in which many home automation devices, home appliances, consumer electronics and communications equipment are interconnected and oriented toward the needs and demands of the user. The interconnection allows the provisioning of new services and assistant functionalities (e.g., monitoring elderly/patient users [1,2]) that go beyond the individual value of the home’s appliances [3].

A main goal of smart homes is to offer a better quality of life for their users in comparison to traditional homes. Everyday activities could become intuitive, enjoyable, convenient, safer, easier, faster and better in many ways [4]. To provide a variety of services to users, as much context information (e.g., location, time, user/device profiles, etc.) as possible should be available. Context-aware services are a key in order to offer an improved range of services. Among context information, user location is crucial for a context-aware service provision system [5], as many smart home services are based on information about the current location of the user, which is why particular attention is being focused on User Location Discovery (ULD).

Conventional ULD systems are not user friendly. They either force a user to carry a device (e.g., a mobile phone or a tag) or they monitor user’s actions via a camera system. This paper considers the following multi-objective research question: how to design a novel ULD system for context-aware services in future smart homes that will (i) respect user’s privacy, (ii) not force users to carry any special device and (iii) be fault tolerant and provide adequate accuracy in localizing users.

The technologies of the Internet of Things (IoT), embedded systems, intelligent devices and machine-to-machine communication are already extending into our daily life. The IoT will introduce more and more sensors into smart homes, creating a new ecosystem with new opportunities for smart home services. With the advent of low-cost, low-power sensors, designing a ULD platform that utilizes sensors already available in smart homes could indeed eliminate the problems associated with current ULD systems.

We propose a sensor network-based and user-friendly ULD system that utilizes different types of inexpensive (mostly already installed) sensing nodes combined with a context broker that uses a fuzzy-based decision-maker. The proposed solution provides a simple, but effective method that meets users’ demands for privacy and comfort. A user does not need to carry a device, and our system does not use sensors (e.g., cameras, microphones) that impose on user’s privacy. Sensors detect the presence of a user and send the context information to a fuzzy-based decision-maker. The decision-maker processes the context information based on fuzzy set theory and makes a decision about the user’s location. We present a use case of our proposed ULD system to illustrate the continuous observation of the same content by means of multiple devices with different resolutions and screen sizes independent of user location. Based on the use case, we define a scenario and evaluate our proposed ULD system.

The rest of the paper is organized as follows. An overview of ULD methods is presented in Section 2, followed by a description of the proposed ULD method in Section 3. The use case, scenario and evaluations are presented in Section 4, and we conclude in Section 5.

## 2. Background

Thanks to the emerging trends of IoT and context-aware services, indoor localization has been the subject of intense research in recent years. Several systems that differ in their technology, localization technique or range were developed for different types of applications [6].

### 2.1. Physical and Symbolic Localization

A ULD system can provide two kinds of information: physical and symbolic [6]. Symbolic location encompasses abstract ideas of where something is, while the Global Positioning System (GPS) can detect physical positions. The resolution of physical positioning systems can have direct implications on the definitiveness of the symbolic information with which they can be associated. For example, knowing where a user is inside a building, to within 10 m, is not very effective at placing that user in a specific room because of the position of walls within that 10-m range. A symbolic location system can provide only very coarse-grained physical locations. A symbolic location system often requires readings or sensors to increase accuracy, such as using multiple overlapping proximity sensors to detect someone’s position within a room. In this paper, we mainly focus on symbolic location.

### 2.2. Cooperative and Non-Cooperative Users

Generally, ULD methods in smart homes can be classified into two groups based on the role of the user [7]: cooperative users and non-cooperative users. In cooperative user methods, the end user actively interacts with the components of the system. Personal wireless devices, such as mobile phones or other dedicated wearable sensors, are usually used by a cooperative user for direct communication with the smart home infrastructure. In such a configuration, the user is most often a mobile node of the network, recognized by the system through the identification of the associated devices. The localization and the behaviour interpretation are based on the processing of data actively exchanged with the wireless devices. These methods use a device for ULD, and so, we can also consider them as Device-Based Localization (DBL) methods.

Unlike cooperative users, with non-cooperative users, no wearable devices are (usually) present, and no direct communication with the system is established by the user. The user is part of the environment instead of being part of the wireless network infrastructure. Therefore, the user behaviour detection depends on the ability of the system to sense the environmental changes and, in particular, the perturbation caused by the user’s presence and movements. We can consider non-cooperative user methods as Device-Free Localization (DFL) methods. DFL is the practice of locating people or objects when no tag or device is attached to the entity being tracked [8]. Two common examples for DFL are camera based and wireless based. Optical camera-based methods do not expect the user to carry a device, but their function is usually very dependent on lighting conditions. High bandwidth requirements and limited view angles are the other major problems with optical camera-based methods. Thermal cameras, a variant of the optical camera, are extremely powerful for many applications. They utilize a directional heat sensor similar to those used for optical sensing. Wireless-based DFL methods [9] enable the users to proceed with their daily activities without having to wear a traceable device. The main principle is the absorption phenomenon of the Received Signal Strength (RSS) of transmitted wireless signals as the human body crosses a transmitter-receiver path. By using transmitter-receiver pairs, the absorption characteristics of a human body exhibit signal patterns that can be used in locating and tracking within a fixed environment.

### 2.3. Related Work

Based on the above discussion, we have studied some important ULD methods and analysed them in Table 1.

A ULD system based on a probabilistic filtering technique (Bayes filter) to estimate and localize a single user in smart homes was proposed by Ballardini et al. [1]. Their proposed ULD method divides the home environment into some zones, and fixed passive motion sensors are installed for user detection. The method can localize a user with sub-room accuracy without asking him or her to carry any mobile device. Different from this work, which is based on one type of sensor (i.e., motion sensor), we propose utilizing different types of sensors that mostly have been already installed in smart homes. This idea gives us this opportunity to detect user location not only based on his or her movement, but also considering his or her other situations (e.g., smoking, sitting, sleeping, washing hand/dishes). In the condition that one type of sensor cannot detect the user, using different types of sensors can increase the accuracy and fault tolerance of ULD systems.

In [10], the authors proposed an approach to detect user motion and location. Based on human kinematics and locomotion phase detection, they determine the spatial position and the trajectory of a reference point on the body. To detect the orientation of user’s body segments and the lower limb joint motions, they ask their users to wear 8 sensors. The sensors are attached on the limbs for motion capturing (i.e., one on the trunk, one on the pelvis, and three for each leg attaching one on the thigh, the shank, and the foot, respectively). They used shoe pad sensors for contact detection. Although their experimental results show good accuracy, forcing users to wear and carry several sensors can be considered as a big limitation and problem.

Wu et al. [11] have presented a Wireless Indoor Logical Localization (WILL) approach. By exploiting user motions from mobile phones, they could successfully remove the site survey process of traditional methods, while still achieving competitive localization accuracy. The rationale behind WILL is that human motions can be applied to connect previously independent radio signatures under certain semantics.

A ULD method that does not need any wireless integrated circuit (IC) tags or target nodes was investigated in [12], which considers the case where the user is walking in a room. The ULD is achieved using a Multiple-Input Multiple-Output Ultra-Wide Band (MIMO-UWB) radar system that measures the propagation channels between the antennas and the human body. The waves reflected by the human body are extracted by using the differences between consecutive snapshots of the impulse response, eliminating the need for any pre-measured room response characteristics. In addition, by using this MIMO radar system, many pairs of propagation channels between the antennas can be measured, leading to a reduction in the effects of clutter, a major cause of errors in radar systems.

Shen et al. presented an idea based on radial distance modulation to detect and locate moving objects from a top view angle [13]. This method has the advantage of directly extracting the information from the moving object’s characteristics and spatial position. Their experiments demonstrate that although the output of Passive InfraRed (PIR) detectors only has two values, zero and one, they can locate a moving object with simple information after modulating and encoding the sensors’ perception area.

Yang et al. [14] proposed a ULD method using PIR sensors. They also utilize an accessibility map to reduce the uncertainty of localization discovery, where the typical PIR-based ULD solutions suffer. The accessibility map represents user habits in smart homes, which also includes geometric and furniture layout information. Particle filtering is used to improve the location accuracy. Their method needs a fine map based on long-term monitoring.

In a nutshell, existing related work mostly relied on user location prediction techniques. These techniques are usually time-processing-consuming and need rather complex algorithms, large-sized databases and creating accessibility maps. In this paper, we propose a simple fuzzy set-based technique that makes real-time decisions to detect user location. To the best of our knowledge, this work is the first effort of using the fuzzy set technique for ULD. Combining a variety of sensor types with a decision-making fuzzy set-based algorithm supports the accuracy of our proposed ULD system in the existence of sensor failure.

In addition, existing related work for ULD systems assumed that their equipment should be installed in smart homes independently. Emerging new technologies, such as IoT and intelligent appliances, are increasing the number of sensors and the complexity of smart homes. Considering this point, different from existing related work, we think about the possibility of using already-installed equipment (e.g., sensors) by emerging technologies (e.g., IoT) to reduce the cost of the proposed ULD system, as well as the complexity of smart homes.

### 2.4. Research Challenges

Future smart homes will need to address some challenges. The most important challenges for ULD systems in future smart homes are: 

**User comfort (ease of use) and simplicity:** One of the most important aspects that will definitely encourage their adoption is simplicity, ease of use and level of comfort. A user-comfortable system should not force a user to carry a device for localization inside a home.

**Privacy and trust:** The preservation of user privacy is crucial for any ULD system. Location information is very sensitive, and the identity of users should only be accessed under special authorization and within the purposes described in the application agreement that should have been agreed upon by the users. Privacy becomes an even more complicated problem for ULD systems that use devices like cameras or microphones for user localization.

**Accuracy:** Localization accuracy is always a basic consideration for smart home applications. It has a direct effect on the performance of a ULD system. Depending on the type of application, the level of the required localization accuracy will vary. For example, for a heating/cooling application, it would be sufficient to know if the users are present and in which room they are located. On the contrary, for senior care and monitoring applications, it is desirable to know the precise location (and sometimes the situation in terms of activity and health) of users at all times, which requires much more accurate mechanisms and more complicated methods.

**Fault tolerance:** It is essential for a system to continue operating properly in the event of the failure of some of its components to assure a reliable operation.

**Multiple-user localization:** It is required to support more than one user. Most of the current DBL and DFL systems cannot support multiple users.

**Latency:** Aligned with the accuracy issue, latency or speed is another major challenge that can be very critical for some applications

**Security:** The development of a secure and trustworthy ULD system that respects privacy concerns is a challenging task, one that is decisive for the adoption of these systems by the public. Security requirements are somewhat aligned with privacy and trust issues. Apart from privacy and trust preservation, the use of secure ULD approaches will avoid undesirable situations, such as theft. Encrypted communications are one of the techniques for providing secure ULD.

**Cost:** Another important metric for users is the cost of ULD systems. Many users do not have the means and/or are not willing to invest a large amount in a ULD system. 

This paper mainly focuses on improving user privacy and comfort while meeting fault tolerance challenges. Acceptable accuracy and cost are also considered as important metrics. Some practical points that we will consider in designing our ULD system follow:

- For user comfort and ease of use, we design a ULD system without forcing users to carry a device.
- Devices that directly affect user privacy, such as cameras and microphones, will not be used in our ULD method, so that user privacy can be assured.
- To keep costs low, we will consider using some pre-installed devices (e.g., sensors already being used by other applications).

## 3. Sensor Network-Based and User-Friendly User Location Discovery

To improve user privacy and comfort, our ULD system is designed to be more user friendly than conventional systems.

**Figure 1:**
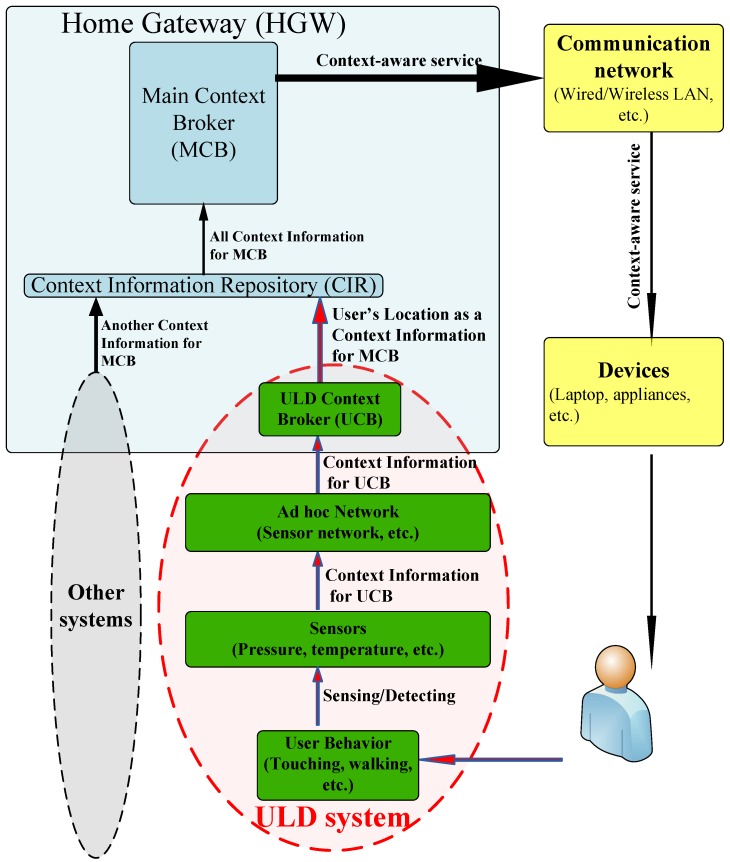
A high-level architecture for supporting context-aware services in future smart homes.

Figure 1 shows a high-level architecture for supporting context-aware services in future smart homes and clearly presents the role of our ULD system in the architecture. Different types of sensors monitor the home environment to detect the user(s). Each sensor that detects a user sends a witness signal (as context information) through a sensor network via heterogeneous interfaces (e.g., ZigBee, Bluetooth, WLAN) to a Home Gateway (HGW).

A ULD Context Broker (UCB) in the HGW processes all of the received context information to detect the current user location. After detecting the location and processing the context information, the user location information is stored in the Context Information Repository (CIR) of the HGW as one part of the context information for a Main Context Broker (MCB). Normally, there are other types of context information in addition to location information. This paper focuses on the ULD system and user location.

Other types of context information and the methods of assembling them are out of the scope of this paper. The MCB takes all of the necessary context information from the CIR and processes it to offer a service (e.g., content delivery, child protection) to the user. Finally, the MCB makes a decision about how it can provide the requested service to the user. The MCB serves the requested service to the appropriate devices for the user through a home communication network. Before designing the user-friendly ULD system, we first conducted a comprehensive study to find some appropriate sensors.

Next, we consider the model to use with the selected sensors, and finally, we propose a UCB with fuzzy-based decision-making to process the context information and perform ULD.

### 3.1. Selecting Appropriate Sensors

**Figure 2:**
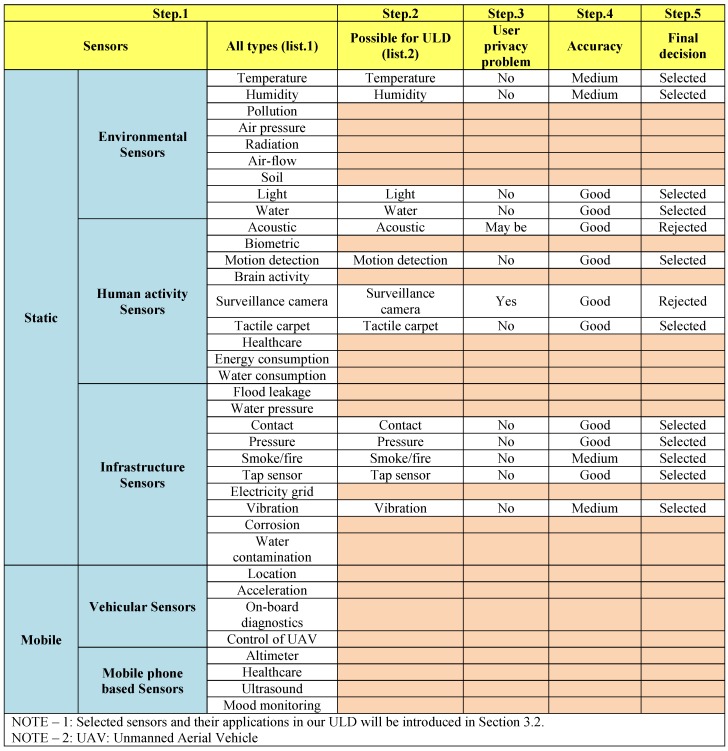
Selecting appropriate sensors for ULD from different types of sensors.

Figure 2 (partly derived from [15]) shows a comprehensive collection of the different types of sensors that may be found in future smart homes for different applications. We used a five-step elimination process to select the most appropriate sensors. First, we found and categorized all of the possible sensors (list.1), and then we selected those sensors that could potentially be used for ULD systems (list.2). In the third step, we evaluated the sensors in list.2 in terms of user privacy. We then assessed those possible sensors for their accuracy and finally selected some sensors that could be useful for our ULD system.

### 3.2. Deployment of Selected Sensors in a Smart Home

**Figure 3:**
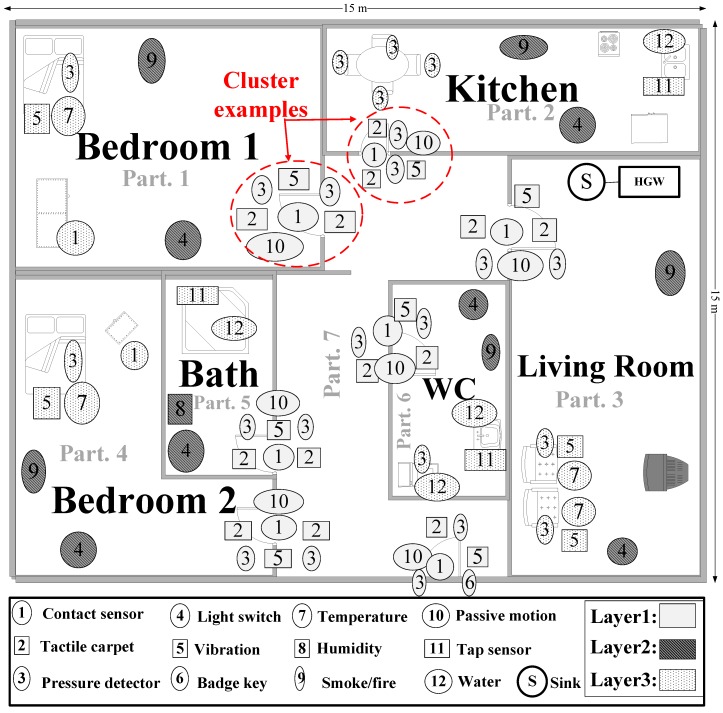
A high-level architecture for the sensor network-based and user-friendly ULD system in future smart homes.

Our proposed future smart homes could consist of an HGW, a sink node and a number of wireless sensor nodes, as well as some partitions (seven partitions from Part. 1 to Part. 7), as shown in Figure 3.

The HGW can be located in any convenient place in the smart home. The sink node is a specially-designed sensor node that has more memory than other sensor nodes and is connected to the sink through a wired or wireless link. The sensor nodes are distributed in the smart home so that the radio coverage of any sensor node covers at least one other node. Whenever each sensor node witnesses an event, it sends a signal to the sink. The sink node collects data from the sender nodes and delivers them to the HGW. All of the sensor nodes can be battery-powered, except the sink node, because the sink node is the most frequently-used node in the network. When deployed, the transmission power of each sensor node is regulated so that its radio coverage is fixed.

Every sensor node is required to monitor its own battery power level and has its own identification (ID) number and location information (i.e., it is located in Part. 1). The sensors we selected and how we used them in our smart home model are described here: 

**Contact sensors (1):** These are electric contacts on doors (including closets, drawers, pantries, etc.), installed on all doors and drawers. When a door (or a drawer) is opened or closed, an event is triggered.

**Tactile carpets (2):** The sensor carpet (mat) design is based on capacitive sensors using low-cost conducting papers, which have sufficient conductivity for most disposable product applications [16]. We place them near each entrance door in order to easily detect a user.

**Pressure sensors (3):** We use pressure sensors for two goals and in two different types of places:

- Near doors for detecting user location and direction: We use pressure sensors to detect a user’s movement direction. Consider a door between two partitions (e.g., Part. 3 and Part. 7 in Figure 3) of a smart home. We put two pressure sensors on two sides of the door (one sensor near the door inside each partition). If the sensor in Part. 3 detects the user and then the sensor in Part. 7, a user is going from Part. 3 to Part. 7. However, if the sensor in Part. 7 detects the user first, followed by the sensor in Part. 3, then that user is going from Part. 7 to Part. 3.
- Under the mattress or chairs to detect if a user is lying in a bed or sitting in a chair: When a user sits or lays on a bed or a chair, the pressure sensor sends signal to the sink. For more accuracy, some other sensors, such as those for vibration and temperature, are used under chairs and beds.

**Smart light switches (4):** Installed in each partition of the smart home, these are useful especially at night. When the user turns the lights on or off, this sensor can send an event or a signal to the sink.

**Vibration sensors (5):** These are fitted to doors and will activate if they receive any kind of bang or severe vibration. We also use them in chairs and beds to detect a user.

**Badge key (6):** Some homes are equipped with a badge key. When a user uses it to come inside his or her home, it sends a signal to the HGW to announce that the user is coming inside his or her home.

**Temperature (7):** Installed in beds and in some chairs to detect user location based on body temperature.

**Humidity sensor (8):** We use a humidity sensor inside the bathroom. When a user takes a shower, the humidity sensor senses a rapid increase in humidity levels and sends a signal to the HGW. It can be installed over a bath or a shower. The humidity level is user-definable.

**Smoke (9):** This sensor is installed in the ceiling of each partition. If a user is smoking, we can easily detect user location based on a signal sent by this sensor.

**Passive motion (10):** Transforms a detection of motion into an electric signal. One passive motion sensor is installed in each partition. It can detect user movement and send a signal to the sink.

**Tap sensor (11):** When this sensor senses the presence of an object (e.g., a user’s hands) in front of the tap, it sends a signal to the solenoid valve to initiate the flow of water. When the object is no longer present, the infrared unit sends an electronic signal to the solenoid valve to terminate the flow of water, usually after a few seconds. Although the main goal of the tap sensor may be hand detection to start and stop the water flow (and avoid wasteful water flow), it also serves as a good user location indicator. When the tap sensor detects a user’s hand, it sends a signal to the sink.

**Water sensors (12):** These will wirelessly activate if water passes over them. We installed them in a bathtub, a kitchen sink and a water closet (under taps). When a user consumes water, this sensor can detect the user action and position.

### 3.3. ULD Context Broker

For future smart homes, we consider a very simple and effective method for evaluating the context information received from sensors. We divide the smart home into different partitions. Each room can be considered as a partition, and we consider the following four detector layers inside each partition (see Figure 3):

**Entrance gate detectors (Layer 1):** This layer is the most important layer of our ULD architecture, as it is designed to detect if a user is coming or leaving a partition. It is equipped with five different types of sensors at the entrance port(s) of each partition to detect a user. Using a variety of sensors offers a very high level of user detection.

**Environment detectors (Layer 2):** These monitor the changes in the environment of each partition, such as sensing smoke to detect user location.

**Infrastructure detectors (Layer 3):** Monitoring infrastructures, such as taps, produces good indications of a user’s location.

**Device detectors (Layer 4):** We do not use this layer here, but we believe electronic devices, such as PCs, laptops and phones, can help us to detect user location. We reserve this layer for our future work. 

Each layer is divided into clusters. Each cluster includes a group of sensors that are located near each other and can detect a user at the same time.

Our proposed UCB is based on fuzzy set theory. We combine the concept of context awareness with fuzzy set theory. The proposed ULD receives context information from different types of sensors and evaluates that data using the fuzzy set theory.

The theory of fuzzy set, introduced by Zadeh [17], is a tool to model uncertainty and for processing vague or subjective information in mathematical models. The notion central to fuzzy systems is that membership values are indicated by a value in the range [0, 1], with zero and one representing absolute falseness and absolute truth, respectively.

Consider a situation that some sensors in different locations simultaneously detect the user. Obviously, the user cannot be present in different locations (e.g., different rooms) at the same time. The fuzzy set theory can help us in these uncertain situations to make appropriate decisions. We consider a fuzzy set *A* that includes all of the possible parts of the smart home, *A* = (p1, p2, ..., pn). Our goal is to find the part where the user is currently located. Based on the concept of fuzzy set, a membership function mA() should be considered for each part of the home. For each partition i, the membership function mA(pi) consists of four factors (i.e., L1, L2, L3 and L4) in a range of [0,1] that relate to Layer 1, Layer 2, Layer 3 and Layer 4 of our layering detector concept. As mentioned above, we reserve L4 for our future work, and so here, we simply define it to offer a comprehensive architecture. A fair cooperation to compute the membership value (function) of smart home partitions can be achieved by adding these factors together. This means that all four layers of each partition (i.e., those in Part. i) will cooperate together to compute a membership value mA(pi). When a sensor detects an event, its situation is changed from passive to active, and it sends a signal to the sink.

The following equation describes the computation of the membership value for the i-th part of a smart home in more detail:

(1) $$mA\left(p_{i}\right)=\delta \times \sum_{L=1}^{4}\left(\phi_{L}\times \frac{\sum_{c=1}^{N_{iL}}\frac{\alpha_{iLc}}{\omega_{iLc}}}{N_{iLAct}}\right)$$

where the denominator ($\omega_{iLc}$) is equal to the total number of sensors in cluster *c* of layer *L* of part i. $\alpha_{iLc}$ is the number of sensors in cluster *c* of layer *L* of part *i* that detect a user. $N_{iL}$ is the number of available clusters in layer *L* of part *i*, and $N_{iLAct}$ is the number of active clusters in layer *L* of part *i* in which currently at least one of their sensors is active. We consider a priority (i.e., ϕ) for each layer. L1 has an important role and, so, has the highest priority (e.g., ϕ = 0.4), and in this version, L4 is considered to have the lowest priority (e.g., ϕ = 0.1). We consider a *δ* factor for mapping the membership value to [0, 1]. When *ω* = *α* for all layers, we will have a maximum value of the membership function (mA(pi) = 1). The part with the highest membership value or mA() will be selected as the most likely current user position.

**Figure 4:**
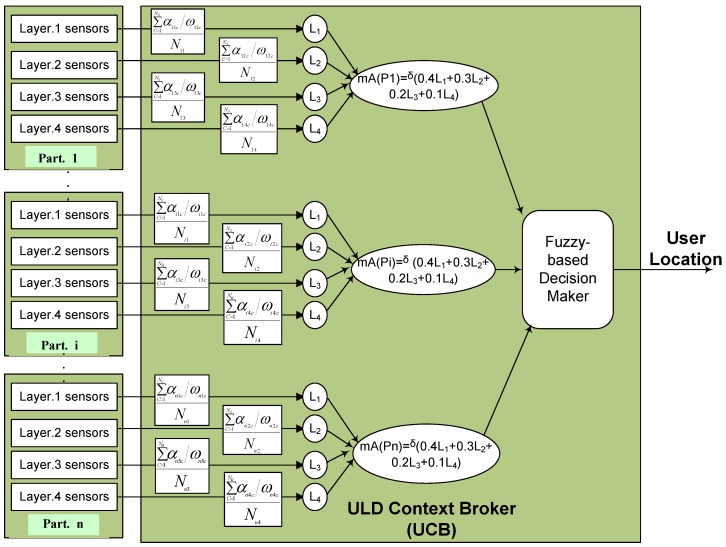
The mechanism for fuzzy-based decision-making in the ULD context broker.

Figure 4 shows the general mechanism of finding user location using the UCB. Sensors in each partition send their context information (if they have any) to the UCB. The UCB then computes membership values for each partition and finally compares all of the membership values and selects the highest one as the user location.

## 4. Performance Evaluation

In this section, we first introduce a use case and define a scenario for evaluating the proposed ULD method. Next, we manually run the scenario to demonstrate the performance of the proposed method. Finally, we simulate the scenario and evaluate the proposed idea based on a series of comprehensive tests.

### 4.1. Use Case and Scenario Example

**Figure 5:**
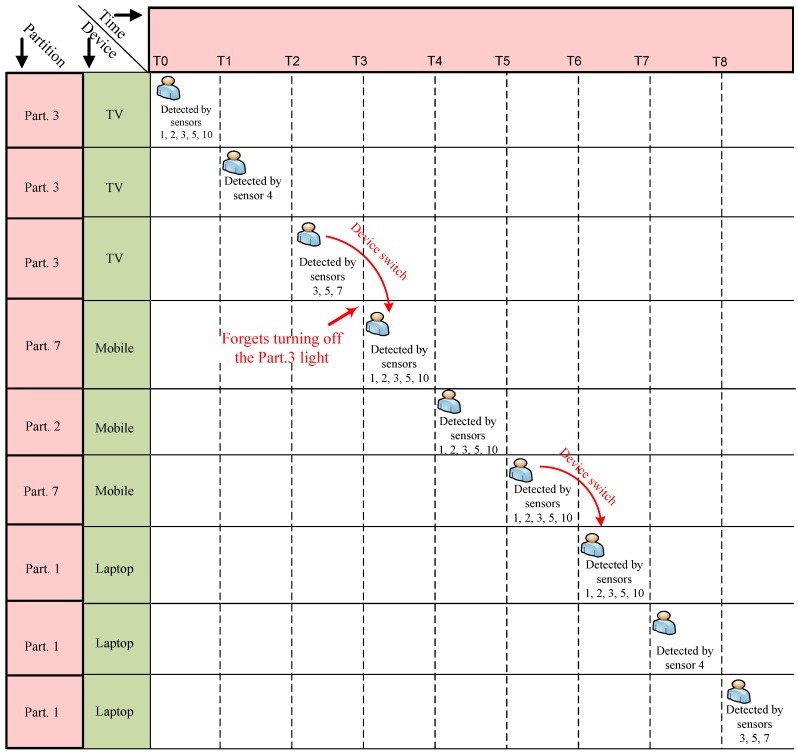
The scenario: Alice’s locations in a smart home.

Continuously watching the same content on a three-screen TV [18] has been considered as one of the most representative context-aware services in future smart homes. In order to watch the same content on several devices continuously, this service should support content mobility among multiple types of screens with different resolutions based on user location in a smart home. It automatically selects the best available device for watching content. Here, we illustrate our ULD system supporting this service in the future smart home environment shown in Figure 5, considering the following scenario.

A user (i.e., Alice) enters her living room at time T0, turns on the light at time T1 and sits on a chair at time T2 to watch content on her TV. At time T3, she leaves the living room (e.g., forgets to turn off the living room’s light and the TV), and at time T4, she goes to the kitchen and wants to continue watching that content on her mobile device. After that, she leaves the kitchen at time T5 and goes to Bedroom 1 at time T6, turns on the light at time T7 and lies on the bed at time T8; watching what remains of the content on her laptop. Figure 5 shows the scenario.

### 4.2. Analytical Model

Here, we manually run the same scenario described above along with our proposed ULD method.

In our ULD system, at time T0, a cluster of L1 layer sensors located around the living room entrance door (Part. 3) detect Alice’s presence and send signals to the sink. At time T1, an L2 sensor (light switch) in the living room detects Alice, and then, a cluster of L3 layer (chair) sensors in the living room detects Alice at time T2. At time T3, a door cluster (L1 layer) of sensors in the living room detects that Alice is leaving the living room (she has forgotten to turn off the living room light). At time T4, she goes to the kitchen (Part. 2) and is detected by the kitchen’s door entrance sensors.

In this situation, the fuzzy-based UCB has two possibilities to select: (i) the living room (that its light is still on) and (ii) the kitchen where a cluster of L1 layer sensors detects Alice. The UCB easily selects Alice’s correct location because, on the one hand, the priority (coefficient) of the L1 layer sensors is higher than that of the L2 layer sensors, and on the other hand, there is just one active sensor (light switch) in the living room, while all (or at least most) of the door cluster of the kitchen’s L1 layer are active. At time T5, the door sensors detect that she is leaving Part. 2 and entering Part. 7.

**Figure 6:**
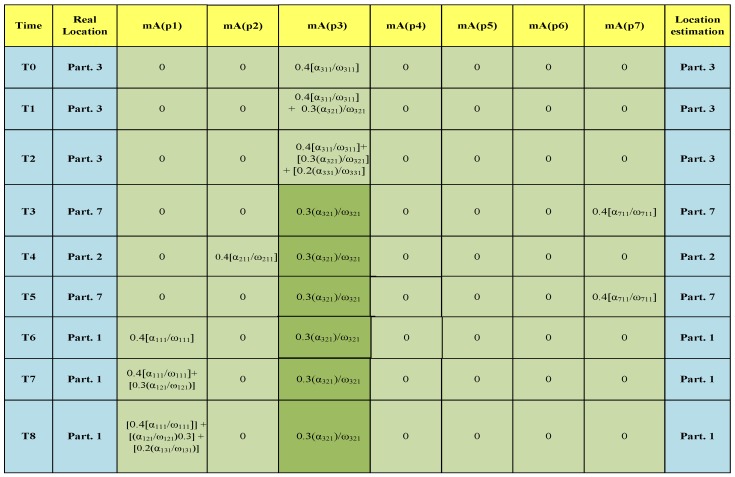
The scenario: detection mechanism.

At time T6, the sensors located around the Bedroom 1 entrance (the L1 layer door cluster sensors of Bedroom 1) detect that she is coming into Bedroom 1 (Part. 1). Then, at time T7, some L2 layer sensors in Part. 1 detect Alice, and she is detected by L3 layer sensors at time T8 (see Figure 5 and Figure 6).

The benefit of our ULD system is that it has the ability to find a user’s correct location even under conditions of abnormal user action, sensor failure or sensor inaccuracy. As Figure 6 shows, after T3, there were two possible candidates for Alice’s location (because she forgot to turn off the Part. 3 lamp). However, the UCB can find the right location of her based on the fuzzy set theory. After just time T3, there was only one active sensor in Part. 3 (light switch of Layer 2).

Therefore, the value for Part. 3 will be $\left(0.3\times \frac{\sum_{C=1}^{N_{il}}\frac{\alpha_{32c}}{\omega_{32c}}}{N_{31}}\right)=\left(0.3\times \frac{\alpha_{321}}{\omega_{321}}\right)$, which is less than the membership value of the parts for all (or most) of the L1 sensors that detect Alice (see Figure 6).

### 4.3. Simulation

To evaluate the performance of our proposed method, we basically simulate the use case scenario in Section 4.1 in the smart home environment shown in Figure 3. We used the DPWsim simulator [19] and conducted four different tests as described below.

To make the simulations more accurate, we consider different rates of sensor error. The sensor error in this simulation occurs when a sensor cannot correctly detect a user’s presence. This error covers sensor inaccuracy, human faults (e.g., forgetting to turn off a light switch) and even some environmental negative effects on sensors (e.g., darkness at night). We also consider sensor failure as another important factor in evaluating the performance of ULD systems. Sensor failure occurs when a sensor is completely out of service (e.g., its battery is dead).

To evaluate the performance of the proposed ULD method, we consider four different tests, as listed below: 

**Test 1: detection accuracy:** Localization accuracy is one of the most important characteristics of each ULD system. Test 1 evaluates the accuracy of our system by considering different levels of sensor detection error rates. This test is considered as a main step to evaluate the performance of our proposed method. The accuracy is indicated as the percentage of times the ULD system detected the correct location (part) of the user in the presence of different rates of sensor errors.

**Figure 7:**
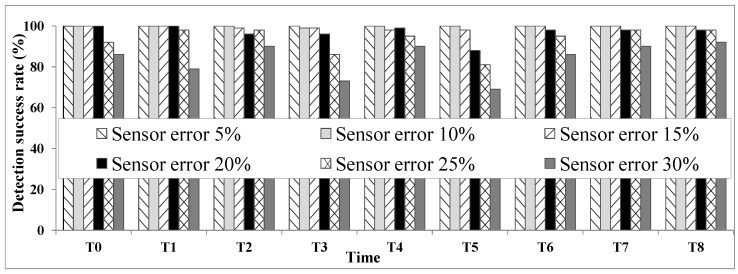
Test 1: detection accuracy.

We considered different types of sensor error rates from a low (5%) to a high rate (30%). Based on the scenario in Section 4.1, we verified the user location at eight time slots. As Figure 7 shows, the system works perfectly in the presence of low sensor error rates. The system even shows an accuracy level of 70% or higher when faced with very high rates of sensor error. In general, for sensor error rates under 20%, the ULD system has a quite acceptable performance, proving both its accuracy and robustness.

**Test 2: fault tolerance:** It is an important issue if a system can work correctly even in the presence of a fault. As mentioned earlier, sensor failure means a sensor becomes completely out of service. Test 2 considers different rates of node (sensor) failure and shows the performance of the proposed system in the presence of different sensor error rates.

We simulated six different rates of node failure ranging from 0% (no fault) to 5%. We also showed the detection rate of a single sensor in different sensor error rates (normal situation). The normal situation can be used as a reference to better understand the effectiveness of our ULD method.

**Figure 8:**
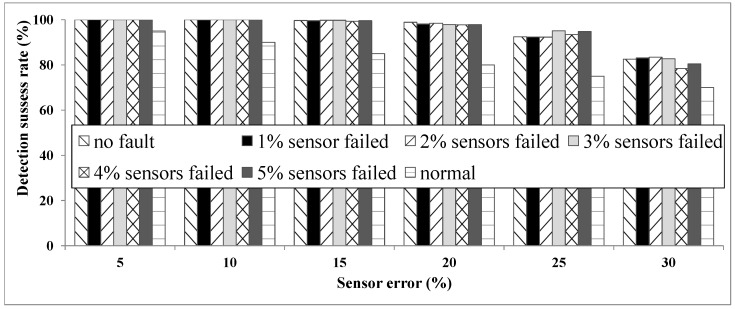
Test 2: fault tolerance.

In Figure 8, we can understand how our system is completely fault tolerant given the low rates of node failure. In fact, we cannot see any notable challenges to the system as the number of node failures is increased. This robustness is partly because the system is based on the functioning of multiple sensors.

**Test 3: relation between the number of sensors and accuracy:** This test shows the relation between the number of sensors and the system’s accuracy. Test 3 also allows us to determine the appropriate number of sensors for the system.

In this test, we increase the number of sensors in each part of the smart home. First, we consider only one sensor for each layer in each part (a total of three sensors). Then, we gradually increased the number of the sensors, up to 30 in each part.

**Figure 9:**
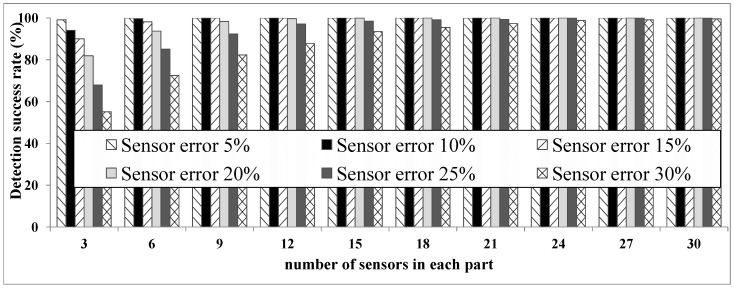
Test 3: relation between the number of sensors and accuracy.

The results of Test 3, shown in Figure 9, show that the number of sensors has a direct effect on system accuracy. Figure 9 shows that increasing the number of sensors increases the system’s accuracy. However, there appears to be a threshold for the ideal number of sensors. Using many sensors above this threshold can increase the complexity of the system and does so with minimal (or no) gain in accuracy.

**Test 4: membership function value monitoring:** The goal of this test is to discover more details about the mechanism of detecting user location using the proposed ULD system. Test 4 was conducted when the user was in Part. 3. By evaluating the amount of the membership function values of different parts of the smart home, we can better understand the system’s accuracy in determining user location.

**Figure 10:**
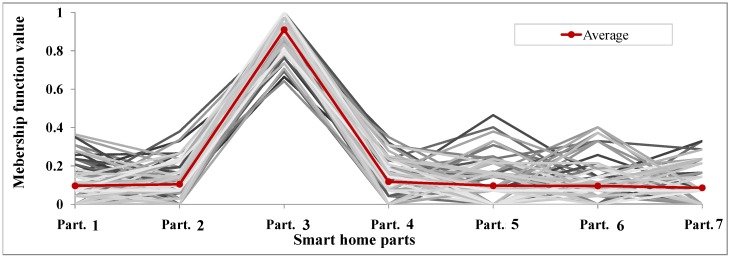
Test 4: membership function value monitoring.

We ran the proposed algorithm to detect the user’s location when the user was in Part. 3. We assumed a sensor error rate of 10%. We repeated the simulation 100 times and took an average. Figure 10 shows the results. We can see that for all 100 simulations, the algorithm could detect the user location correctly, as the membership function amount in Part. 3 is sharply higher than for other parts. This test demonstrates the accuracy of the proposed ULD method in correctly detecting a user’s location.

## 5. Conclusions and Future Work

User location information will play an important role in many context-aware systems designed for future smart homes. We have first discussed the limitations/challenges of current ULD systems. We then mainly focused on improving user privacy and comfort while meeting fault tolerance challenges. Two other important challenges of detection accuracy (i.e., finding the correct location (part) of the user) and cost were also considered in this paper.

To address the above-mentioned challenges, this paper proposed a ULD system without forcing users to carry a device to support user comfort and ease of use. The proposed ULD system also has not used any device (e.g., camera and microphone) that directly affects user privacy. Using some pre-installed devices (e.g., sensors already being used by other applications) kept the cost of the proposed system low. Regarding fault tolerance, the proposed ULD system uses different types of sensors, which give the ability of detecting the user in the presence of sensor failure. For detection accuracy, we have proposed a simple, but effective context broker based on the fuzzy set theory. We claimed that our proposed ULD system, combining different types of sensors along with the fuzzy-based broker, can detect user location even in the presence of sensor/user faults. To prove the claim and show the feasibility of our proposed system, a use case scenario has been presented. The defined scenario, first, has been manually analysed and, then, simulated. Both analytic and simulation evaluations showed the effectiveness of the proposed ULD method in aspects such as detection accuracy and fault tolerance.

The proposed system will definitely involve some important points as future work, such as: (i) a way of incorporating the ULD system with other smart home systems. In other words, how the ULD system could share a very similar infrastructure (e.g., sensors) with other applications (systems) in smart homes (e.g., sensor network virtualization is a technology that can potentially enable this sharing); (ii) in our fuzzy-based decision-maker, the method of clustering sensors and also computing the optimal amount of priority (i.e., ϕ) for each layer play critical roles in the performance of the ULD systems. Selecting large or very small difference between the priority amounts of layers can decrease the accuracy of the ULD system; (iii) supporting location discovery for more than one user. At the current time, the DFL approaches (i.e., do not force users to carry/wear any device) can only be used for applications where a single user is present in the environment (e.g., a single elderly person who lives alone in his or her home). Until now, there has not been any appropriate solution to support multiple users for DFL methods. Finding solutions to extend this type of ULD system to support multiple users is an important future work objective.

In addition to the typical limitations of DFL methods (i.e., one cannot ask users to carry/wear any device), our ULD method should not use any device/technique that may violate users’ privacy (e.g., using a camera). For future work, we plan to find a solution for our ULD method to detect and identify multiple users based on finding various user contexts (e.g., user height, weight and even foot size). In the first step, we will study easily available and inexpensive PIR sensors. The analogue output signal of PIR sensors may include more contexts beyond only user presence, such as the distance of the user from the PIR sensor, the velocity and direction of the user movement and even body shape and gait. These contexts may help us to find an appropriate way to identify and support multiple users.

## Figures and Tables

**Table 1:** Different User Location Discovery (ULD) methods. DFL, Device-Free Localization; PIR, Passive InfraRed; DBL, Device-Based Localization; MIMO-UWB, Multiple-Input Multiple-Output Ultra-Wide Band.

Source	Type	Detection Level	Application	Equipment	Technique(s)	Algorithm(s)
[1]	DFL	Symbolic	Localization and tracking	ZigBee devices equipped with PIRs	Probabilistic filtering, motion detection	Bayes-based algorithm
[10]	DBL	Physical	Localization and tracking	Wearable tags, contact sensors	Joint Motion Tracking, Continuous Root Location Update	Sensor mapping calibration, Footprint skeleton calibration
[11]	DBL	Symbolic	Localization	Mobile phone and access point(s)	Logical localization (WiFi fingerprints and user movement)	Skeleton mapping and branch knot
[12]	DFL	Physical	Localization	Multiple antennas (MIMO-UWB system)	Measuring the propagation channels between the antennas and the human body	Distance estimator (time difference between sending the pulse and reception of the echo)
[13]	DFL	Physical	Localization and tracking	Wireless pyroelectric infrared sensors	Motion detection	Distributed localization
[14]	DFL	Physical	Localization	PIR sensors	Map-based localization, Bayesian and particle filtering	Data fusion by particle filters
